# Who and How Should Prescribe and Conduct Exercise Programs for Pregnant Women? Recommendations Based on the European Educational Standards for Pregnancy and Postnatal Exercise Specialists

**DOI:** 10.34763/devperiodmed.20182202.107112

**Published:** 2018-06-30

**Authors:** Anna Szumilewicz

**Affiliations:** 1 Department of Fitness and Strength Conditioning, Gdańsk University of Physical Education and Sport, Gdańsk, Poland

**Keywords:** education, exercise, exercise specialists, healthcare providers, prenatal care, pregnancy

## Abstract

Extensive scientific evidence has shown that prenatal physical activity is a prerequisite for the proper course of pregnancy, fetal development, labour and delivery, and the postpartum period. In 2015, the American College of Obstetricians and Gynecologists published a breakthrough statement that inactivity in pregnancy is risky behaviour. The aim of this paper is to provide answers to the questions concerning who and how should prescribe and conduct exercise programs for pregnant women.

Close cooperation between the woman, her obstetric care provider and exercise specialists is necessary to promote exercise in pregnancy. Obstetricians should carefully evaluate women with medical or obstetric complications before starting the exercises. They should also provide women with general information about the benefits of prenatal exercise and the risks of physical inactivity. On the other hand, the design and implementation of the exercise program are tasks for exercise professionals, preferably ones qualified according to the European educational standards for Pregnancy and Postnatal Exercise Specialists.

Both empirical observation and scientific research confirm the need to educate obstetric care providers, exercise professionals, and both pregnant women and their families about prenatal physical activity. They all require constantly updated information on how to use exercise to stimulate the positive development of pregnancy, ensure the greatest possible well-being for the future mother, and prepare her for childbirth and the postpartum period. These educational activities should be evidence-based. They must not perpetuate unfounded opinions, often harmful to the woman’s health, on what she should or should not do while exercising during pregnancy. The presented update underlines some pertinent recommendations in this area.

## Introduction

Ample scientific evidence showing that prenatal physical activity is a prerequisite for the proper course of pregnancy, fetal development, labour and delivery, and the postpartum period has dramatically changed the approach to this issue [1, 2]. In the nineteenth century it was recommended that exercise during pregnancy should be avoided in order to conclude it safely [3]. In 2015 the American College of Obstetricians and Gynecologists (ACOG) published a breakthrough statement that the lack of physical activity in pregnancy is risky behaviour [4]. This thread, discussed among others by Professor Katz in the previous issue of the Journal [5], raises the following important questions: to what extent should a pregnant woman be active and who and how should prescribe and supervise an exercise program for her?

According to the ACOG recommendations, obstetrician-gynaecologists and other obstetric care providers should encourage their patients to continue or commence exercise as an important component of optimal health [4]. Fulfilling this recommendation by the medical community seems to be particularly important. Jukic *et al*. [6] observed that advice given by health professionals is associated with a high volume of recreational physical activity performed by their patients. Pregnant women have regular medical appointments, which creates an excellent opportunity for the regular promotion of physical activity. Unfortunately, according to research conducted on a population of 2,852 Polish women in 2011, only 1% of our obstetricians encouraged women to increase prenatal physical activity and 56% did not even mention the need for exercise [7]. This phenomenon is not specific solely for Poland. Similarly alarming results have been observed, e.g., in Norway [8], Germany [9], and South Africa [10].

In order to promote exercise in pregnancy, close cooperation between the woman, her obstetric care provider and exercise specialists is necessary. For example in Canada, the official guidelines on exercise in pregnancy suggested that ’PARmed-X’ (Physical Activity Readiness Medical Examination), a guideline for health screening prior to participation in a prenatal fitness class or other exercise, should be a standardized tool to be included in such collaboration [11, 12]. A clear division of tasks is also necessary. Obstetricians should carefully evaluate women with medical or obstetric complications before starting their exercise program [4]. They should also provide them with general information about the benefits of prenatal exercise and the risks of physical inactivity. In contrast, the design and implementation of the exercise program are tasks for exercise professionals. South African researchers, Watson *et al*. [10], observed that only 24% of health practitioners referred pregnant women to exercise specialists.

Many years of experience gathered in the implementation of experimental research on pregnant women allowed me to observe the increasing openness of healthcare providers to prenatal exercises. These observations also gave rise to assumptions that obstetricians would more willingly and more often encourage women to exercise if they were assured that these exercises were conducted by professionals well prepared for this purpose.

## European standards for pregnancy and postnatal exercise specialists

At the international level an attempt to discuss how to prepare professionals to plan and conduct exercises for pregnant women was undertaken during the 5^th^ International Standards Meeting in Amsterdam in 2014, organized by EuropeActive (the former European Health and Fitness Association). The result of this meeting are European, evidence-based educational standards for Pregnancy and Postnatal Exercise Specialists, developed by 25 experts from 14 countries [13]. It is the first document of this type specifying the learning outcomes necessary for the design and delivery of a prenatal exercise program (Box 1) which is set within the concept of the European Qualifications Framework. The professional preparation of specialists who conduct exercise classes with pregnant women according to these standards is to confirm the credibility of exercise professionals and increase the trust in them among the medical community.

## Why is proper programming of prenatal physical activity so important?

In our previous review on the influence of prenatal exercise on the course of labour and delivery we concluded that non-programmed, spontaneously undertaken physical activity in pregnancy, although still safe for the mother and child, is not always reflected in an easier and less medically affected childbirth [14]. The effectiveness of the training program is determined by the appropriate interaction of its major components: intensity, duration, frequency, and type of exercise suitable for the aim of the program [15]. Even such small differences as exercising twice or three times a week can change the body’s response. For instance, in an experimental study we observed that the number of sessions performed during an 8-week prenatal exercise program was significantly related to maternal irisin serum concentration [16]. Irisin is an exercise-inducible myokine which may be a useful biomarker in pregnancy to predict the development of gestational diabetes mellitus [17]. Thus, any strategy to obtain its favourable level should be considered, also through the proper design of a prenatal exercise program.

In working with a pregnant woman, one should make sure that the exercises address her special needs as an expectant mother. Due to continuous morphological, biomechanical and physiological changes in the woman’s body, the selection of exercises requires specialist competences. Despite the significant usefulness of the official guidelines on physical activity in pregnancy, such as the ACOG recommendations, they do not provide an exhaustive answer regarding the content of the prenatal exercise program [18].

**Box 1:** The content of the European educational standards for the Pregnancy and Postnatal Exercise Specialist. Adapted from [13].

10 core knowledge areas and skills for the EuropeActive Standards for the Pregnancy and Postnatal Exercise Specialist
• Role and professional development of the Pregnancy and Postnatal Exercise Specialist
• Morphological, physiological and biomechanical adaptations during pregnancy and postpartum
• Psychosocial aspects of exercising during pregnancy and postpartum
• Basic nutritional rules and other aspects of healthy lifestyle related to pregnancy and postpartum
• Potential benefits of exercise during pregnancy and postpartum
• Health issues and safety considerations related to pregnancy and postpartum exercise
• Health and fitness assessment in pregnant and postpartum women
• Prescription, implementation and adaptation of exercise for pregnant and postpartum women
• Specific exercises related to childbirth and motherhood
• Postpartum exercise and health-related issues

### The essential elements of prenatal classes

To stimulate the positive development of pregnancy, provide the greatest possible well-being for the future mother, and prepare her for childbirth and postpartum, each exercise session should include several elements (Table I) [18]. Each of the elements is focused on a different goal. Prenatal aerobic exercises may support, e.g., the prevention of excessive weight gain and obesity [19], gestational diabetes mellitus [20] and gestational hypertensive disorders [21]. Resistance exercises (including abdominal and pelvic floor muscle exercises), postural, neuromotor and stretching exercises prevent or reduce typical pregnancy ailments. Through proper exercises, pregnant women can reduce, e.g., the frequency and severity of urinary incontinence [22] and back pain [23]. The most beneficial effect on maternal health seems to be induced by a combination of aerobic and resistance exercises [24, 25]. According to the global trend of promoting vaginal birth, preparation for labour and delivery, which includes breathing exercises, relaxation techniques, birth visualization (conceptualization), and the significance of birth position(s) is recommended as well [26, 27].

**Table I:** The recommended structure of a 60-90-minute exercise session for women with uncomplicated pregnancy.

Recommended physical activity elements	Recommended time (minutes)
• Warm-up	7-10
• Aerobic exercise, e.g., low or high-low impact aerobics, walking or jogging on a treadmill, stationary cycling	15-20
• Resistance exercises (including abdominal muscle exercises), and postural and neuromotor (e.g., body balance) exercises	10-15
• Stretching exercises	5-10
• Pelvic floor exercises	5-10
• Cool down and preparation for birth exercises, e.g., birth position and breathing exercises	5-10
• Relaxation and visualization of pregnancy and childbirth	5-15

### The most common mistakes in the planning of prenatal physical activity

#### Waiting until the end of the first trimester

For the best health benefits, physical activity should be undertaken continuously for several months before conception, and then throughout pregnancy and the postpartum period. Unfortunately, for the first few weeks after becoming pregnant, many women stop exercising or significantly limit physical activity due to fear of miscarriage. Obstetricians often wait until the end of 12 or 13 weeks of gestation to give their patients permission for exercise. Although the risk of spontaneous abortion in the first trimester is about 10% [28], it is not scientifically justified to think that the reason for such an outcome would be the bounces associated with running or jumping, or the physical effort related to physical activity. According to the data available, neither will the continuation of recreational exercise increase the risk of miscarriage, nor will its interruption lower it [29]. Research indicates that there are no significant differences in the early miscarriage rates between recreational runners, aerobics participants, and physically active, fit controls [30]. Therefore, for the alleviation of pregnancy-related discomforts, prenatal physical activity should be undertaken or continued from the day of conception.

#### Considering the intensity of exercise at a heart rate above 140 as dangerous

For the majority of pregnant women moderate intensity of physical activity is recommended [4, 5]. Nevertheless, a too low intensity may be insufficient to stimulate the cardiopulmonary system [31] and thus may not produce the desired health effects. This aspect is particularly important for women with high levels of exercise capacity who were accustomed to participating in high-intensity classes before pregnancy. Although an upper level of safe exercise intensity has not been established, according to the ACOG [4] women who were regular exercisers before pregnancy and who have uncomplicated, healthy pregnancies should be able to engage in high-intensity exercise programs, such as jogging and aerobics, with no adverse effects.

The most commonly used tools to monitor prenatal exercise intensity are the scale of Rate of Perceived Exertion (RPE) and the “talk test” [4]. Because heart rate values are usually higher in pregnancy and more changeable under the stimulation of exercise, using them requires experience and some adaptations. Based on peak exercise tests, Canadian experts recommended exercise heart rate ranges (but not maximum heart rate limits) for various groups of pregnant women (Table II). When referring to these guidelines, one should negate the alleged opinion that there is a necessity to maintain the intensity of exercises below the level of 140 heart rate, still quite common also in the Polish society.

**Table II:** Heart rate ranges for various groups of pregnant women according to PARmed-X for Pregnancy [12].

Maternal age (years)	Fitness level or BMI	Heart rate range (beats/minute)
Less than 20	---	140-155
20-29	Low	129-144
	Active	135-150
	Fit	145-160
	BMI > 25 kg/m^2^	102-124
30-39	Low	128-144
	Active	130-145
	Fit	140-156
	BMI > 25 kg/m^2^	101-120

#### Avoiding abdominal exercises

Although abdominal muscle exercises give obvious health benefits including maintaining the correct posture, in pregnancy their performance is often surprisingly and unjustly forbidden. As a combined result of the growing uterus, changes in the curvature of the spine, and the effects of pregnancy hormones, almost 70% of pregnant women experience lower back pain [32]. To minimize this risk, the ACOG recommends the strengthening of abdominal and back muscles [4]. Strong abdominal muscles also play an important role in the voluntary pushing mechanism triggered during the final phase of vaginal delivery. One can encounter groundless opinions that strong abdominal muscles can lead to miscarriage or a more painful delivery. So far, no studies have been published that could confirm the allegedly negative impact of abdominal muscle exercises on the course of pregnancy, childbirth, or child development.

Moreover, both before and during the exercise program, pregnant women should control the condition of their abdomen in terms of *diastasis recti abdominis*. This Latin-derived term denotes the separation of the two rectus abdominis muscles along the white line, a condition present to some extent in the third trimester in 66 to 100% of women [33]. If diastasis recti is confirmed, some modifications to the exercise taking into account the biomechanics of the abdominal muscles are to be made. The etiology of this connective tissue impairment is not clear. In the light of the available scientific evidence it is groundless to maintain that intense exercises strengthening abdominal muscles before conception or during pregnancy increase the risk of abdominal muscle separation.

#### Spreading unjustified opinions about exercises during pregnancy

It is still true that other unfounded ideas are transmitted from generation to generation. The need to eliminate running or jumping during pregnancy is one of them. It is an unsubstantiated belief that such activities can lead to miscarriage or premature labour. Certainly, with the development of pregnancy and the growing uterus, it may be necessary to limit high-impact movements. Potentially, they may cause unpleasant bouncing of the pregnant belly or enlarged breasts, aggravate urinary incontinence, or intensify lower back pain. However, the decision to eliminate high-impact movements should be based on the assessment of the well-being of each woman and not be a general rule. Similarly, in most cases there is no need to reduce the training load, for instance in the form of lighter dumbbells or lessened resistance on exercise machines, just because female exerciser is pregnant.

Some women are afraid to raise their arms above their heads for fear of miscarriage. This, again, is an unfounded opinion which unnecessarily limits the range of exercises during classes. For a complex exercise program, one should incorporate upper-body movements to activate the whole body, increase the exercise intensity, and improve coordination. Likewise, there is no justification for avoiding frequent changes in direction in pregnancy or making turns, for instance during aerobics or dance classes. Although the centre of gravity of the body, as well as balance and coordination all change during pregnancy, there is no scientific evidence that they increase the risk of collapse or collision during exercise classes compared to the general population.

#### The biggest mistake – the unwarranted recommendation for bed rest or complete prohibition / strong limitation of exercise during pregnancy

ACOG experts warn that patients who are prescribed prolonged bed rest or restricted physical activity are at risk of venous thromboembolism, bone demineralization, and deconditioning [4]. Several reviews have determined that there is no credible evidence to prescribe bed rest in pregnancy for the prevention of preterm birth or improvement of perinatal outcomes [34, 35]. The ACOG provides a list of absolute and relative contraindications to prenatal physical activity, which should be considered before starting an exercise program. However, most women can continue their physical activity, sometimes after only minor adaptations to pregnancy. Unfortunately, research shows that medical personnel often remain conservative and do not support prenatal exercises. In one study, circa 11% of them declared that pregnant women limit their physical activity without any medical reason [7].

## The need for further education for obstetric care providers, exercise professionals, pregnant women and their families

In an epidemiological study, Polish women admitted that the lack of knowledge on how to do sports during pregnancy was one of the main reasons why they had limited their physical activity [7]. In order to better prepare obstetricians, midwives, and exercise professionals to answer questions asked by pregnant women, the above care providers must be well educated in this topic themselves. Malta *et al*. [36] indicated that after an educational intervention health care professionals were almost three times more likely to give their pregnant patients proper guidance regarding leisure-time walking compared to the control group without educational backup. Clearly exercise specialists also require education.

The above studies prove that educational activities must be systemic. In addition to the development and implementation of the Educational standards for the Pregnancy and Postnatal Exercise Specialist in Europe, work is under way in Poland on a system of qualifications for pre- and postnatal physical activity. In this proposal, qualifications at levels 1 and 2 are directed at obstetricians and midwives to confirm their being prepared for the implementation of simple tasks, such as directing a woman to appropriate activities, recognizing contraindications to physical exertion, recommending simple exercises, etc. The qualifications at level 3 and higher have been developed for exercise professionals and cover specialized knowledge and skills to design and conduct pre- and postnatal exercise programs.

## Conclusions

Both empirical observations and scientific research confirm the need to educate the public about prenatal exercises. Health and exercise professionals together with the families of pregnant women should support them in starting or continuing their physical activity. Due to common unjustified opinions about the supposedly harmful effects of exercise on the course of pregnancy, this task may be difficult. The correct allocation of assignments is key here: from proper general and obstetric health screening to encouraging women to physical activity by obstetric care providers, to the design and implementation of prenatal exercise programs under the supervision of exercise specialists, preferably qualified according to international educational standards.
